# Targeting multimorbidity: Using healthspan and lifespan to identify biomarkers of ageing that pinpoint shared disease mechanisms

**DOI:** 10.1016/j.ebiom.2021.103364

**Published:** 2021-05-06

**Authors:** Joris Deelen

**Affiliations:** a Max Planck Institute for Biology of Ageing, Cologne, Germany; b Cologne Excellence Cluster on Cellular Stress Responses in Ageing-Associated Diseases (CECAD), University of Cologne, Cologne, Germany

Advancing age is the major risk factor for chronic diseases, such as cardiovascular disease, type 2 diabetes, cancer and Alzheimer's disease. The rising number of older individuals is thus resulting in an increased burden on our healthcare system. Instead of tackling each of these age-related diseases one by one, focused approaches aimed at the identification of shared disease mechanisms, that can ideally be targeted using lifestyle and/or pharmacological interventions, could be more productive. But, how do we identify the vulnerable people in the population that are at high risk for developing (multi)morbidity? This is a question that the research community tries to answer by looking for so-called biomarkers of ageing, i.e. markers that are predictive of age-related morbidity and mortality. When they are successfully identified, such markers can be used as surrogate endpoints in clinical trials or intervention studies that are aimed at improving general health.

According to the criteria of the American Federation for Aging Research (AFAR) a perfect biomarker of ageing should; (a) outperform chronological age in predicting age-related disease and mortality, (b) be simple (i.e. accurate and reproducible) and inexpensive to test without harming the test subject, and (c) work in both humans and model organisms to make findings directly translatable [1]. Several potential biomarkers of ageing have been proposed [2], including clinical markers involved in physiological (e.g. fasting blood glucose, glycated haemoglobin, and blood lipids) and immune function (e.g. C-reactive protein), but, thus far, none has met all the proposed AFAR criteria.

Many of the proposed biomarkers have shown to be associated with the risk of specific age-related diseases, e.g. an imbalance in fasting glucose or blood lipids has been associated with the onset and progression of type 2 diabetes or cardiovascular disease, respectively. However, it is still unclear if these biomarkers are able to predict healthspan. Healthspan is commonly defined as the number of years lived in good health, i.e. with absence of major chronic diseases and disabilities of ageing [3]. A longer healthspan will thus reflect an increased protection against multiple age-related diseases at once.

In this issue of *EBioMedicine*, Li and colleagues [4] studied the predictive value of commonly used clinical biomarkers of ageing using healthspan and lifespan as outcomes. To this end, they used a large and relatively healthy Swedish cohort for which extensive long-term longitudinal follow-up data on disease diagnosis and mortality was available. As the definition of healthspan they used the number of years lived with absence of major chronic diseases. The authors focused their efforts on ten clinical biomarkers involved in glycaemic control, lipid metabolism, inflammation, and haematological function. The majority of these biomarkers were significantly associated with healthspan and/or lifespan in their cohort, which is not surprising given their known roles in the diseases that are mainly contributing to decreased healthspan, such as type 2 diabetes (biomarkers involved in glycaemic control) and cardiovascular disease (biomarkers involved in lipid metabolism). However, the novelty lies in the fact that the study showed that most of the healthspan-associated biomarkers, with the exception of the ones involved in glycaemic control, seem to exert their effect by influencing multiple age-related diseases at once, providing evidence that these diseases have shared mechanisms that can be targeted to improve general health. Moreover, the polygenic score analyses show that the large majority of the observed associations are likely caused by environmental (i.e. non-genetic) effects. This provides further evidence that lifestyle interventions targeting environmental factors, such as dietary interventions and increasing physical activity, can be used to improve healthspan [5], without the need for pharmacological intervention [6].

A disadvantage of using the earliest onset of a major chronic disease as the end of healthspan, as was done in the current study, is that there is an underrepresentation of late-onset diseases, such as Alzheimer's disease. Hence, an increase in healthspan does not mean that individuals are also protected against such diseases. Future studies should thus try to improve the definition of healthspan to better incorporate the dynamic aspect of changes in health throughout life [3].

Recent efforts have focussed on the identification of novel biomarkers of ageing using omics-based measurements, i.e. epigenomics, transcriptomics, proteomics, and metabolomics [7], given that commonly used clinical biomarkers show limited predictive power in the age group where it matters most, i.e. above 60 years of age [8]. Some of the identified omics-based biomarkers have already shown to be better predictors of lifespan than the well-established clinical biomarkers of ageing, especially at higher ages [8], [9], [10]. Hence, it will be interesting to apply the methodological framework described in this proof-of-principle study by Li and colleagues [4] to determine the predictive value of these omics-based biomarkers on healthspan (and lifespan). The best (independent) omics-based biomarkers of ageing can subsequently be developed further so they can be incorporated in ongoing clinical studies to amend or replace the commonly used clinical biomarkers with the goal to identify and treat vulnerable individuals before the occurrence of (multi)morbidity.

## Declaration of Competing Interest

The author has no conflicts of interest to disclose.
